# Perspectives of older people with uncontrolled type 2 diabetes mellitus towards medication adherence: A qualitative study

**DOI:** 10.1371/journal.pone.0289834

**Published:** 2023-08-10

**Authors:** Sathma Upamali, Sarath Rathnayake

**Affiliations:** 1 Department of Pharmacy, Faculty of Allied Health Sciences, University of Peradeniya, Peradeniya, Sri Lanka; 2 Department of Nursing, Faculty of Allied Health Sciences, University of Peradeniya, Peradeniya, Sri Lanka; Cukurova University: Cukurova Universitesi, TURKEY

## Abstract

**Background:**

Better medication adherence among people with diabetes mellitus was found to be associated with improved glycaemic control. However, medication non-adherence is a significant concern in older people with uncontrolled type 2 diabetes mellitus.

**Purpose:**

To explore the perspectives of older people with uncontrolled type 2 diabetes mellitus towards medication adherence.

**Design:**

A qualitative descriptive exploratory study.

**Methodology:**

A purposive sample of older people with uncontrolled type 2 diabetes mellitus living in the community was recruited. Snowball sampling was applied in community recruitment. In‐depth telephone interviews were conducted using a semi‐structured interview guide. Interviews were transcribed verbatim. Thematic analysis was used in data analysis. The consolidated criteria for reporting qualitative research (COREQ) guidelines were followed.

**Results:**

The emerged six themes were: (a) impact of knowledge, attitudes and practices on medication adherence, (b) treatment-related barriers to medication adherence, (c) impact of age-related changes on medication adherence, (d) person-related barriers to medication adherence, (e) impact of COVID-19 on medication adherence and, (f) role of support systems in medication adherence. Knowledge of the disease process and medications, attitudes towards medication adherence, the practice of different treatment approaches, self-medication and dosing, negative experiences related to medications, polypharmacy, changes in lifestyle and roles, the influence of work-life, motivation, negligence, family support, support received from health workers, facilities available and financial capability are the main factors influence medication adherence. Age-related memory impairment, visual disturbances and physical weaknesses affect medication adherence in older people. Additionally, COVID-19-related guidelines imposed by the government and healthcare system-related issues during the COVID-19 pandemic also affected medication adherence.

**Conclusion:**

Adherence to medications among older people is hampered by a variety of factors, including their knowledge, attitudes and practices, person and treatment-related factors and age-related changes. The COVID-19 pandemic has brought additional challenges. Individualised patient care for older people with uncontrolled type 2 diabetes mellitus to improve medication adherence is timely. Strengthening support mechanisms for the above population is essential.

## Introduction

Diabetes mellitus (DM) is a chronic and non-communicable metabolic disorder characterised by hyperglycaemia. It is considered a global health emergency in the 21^st^ century [1]. Uncontrolled long-term hyperglycaemia leads to numerous complications such as cardiovascular disease, diabetic nephropathy and diabetic retinopathy [2]. The global prevalence of adults (aged 20–79 years) with DM (9.3% in 2019) will increase to 10.2% in 2030 and to 10.9% in 2045 [3]. In 2017, there were approximately 5 million deaths in the age group of 20–99 years globally due to DM [4]. The estimated worldwide direct healthcare expenditure on DM was 760 billion U.S. dollars in 2019 [5]. Consequently, it has become an important global public health concern with a significant social, financial and healthcare system burden [6].

Population ageing, i.e., the shift in the distribution of the world’s population towards older ages, is a global phenomenon with significant welfare and healthcare system challenges [7]. Globally, DM, particularly type 2 diabetes mellitus (T2DM) is one of the most prevalent chronic diseases among older people [7]. Sri Lanka is one of the fastest ageing countries in the developing world [8], and DM is among the most common non-communicable diseases among older people in Sri Lanka [9]. About 1.5 million adults in Sri Lanka are suffering from diabetes [10]. By 2030, the prevalence is expected to rise to 2.1 million [10].

Medications play a vital role in mitigating symptoms and preventing complications of DM. Besides, medication adherence is essential in improving disease control and preventing related health complications [11]. Medication adherence in long-term therapies is defined as “the extent to which a person’s behaviour (taking medication, following a diet, and/or executing lifestyle changes), corresponds with agreed recommendations from a health care provider” [12]. Evidence indicates that better adherence to an anti-diabetic medication regimen is associated with better health outcomes; for example, improved glycaemic control and reduced complications [11, 13]. Moreover, recent studies report that better adherence is associated with decreased healthcare resource utilisation, decreased hospitalisation and improved quality of life of people affected [11, 13].

The management of DM in older people is challenging [14] and medication non-adherence has become a serious and common problem in this population [15]. Consequently, improving medication adherence is essential. However, previous literature focused on patients with diabetes in general [16–19], but little or no attention was paid to older people with uncontrolled T2DM. Therefore, understanding the underlying reasons for non-adherence is essential for establishing interventions and policies to improve medication adherence among older patients with DM. Therefore, this study aimed to explore the experiences and barriers concerning medication adherence among older people with uncontrolled T2DM in Sri Lanka.

## Methodology

### Study design, setting and participants

A qualitative descriptive exploratory study was undertaken. A purposive sample of 14 older people with uncontrolled T2DM participated in this study. Six participants who followed the Diabetic Clinic, Teaching Hospital, Peradeniya, Sri Lanka and eight patients from the community were recruited. In community recruitment, the snowball sampling method was used. In this study, participants aged 60 years and over who were diagnosed with DM by a physician with uncontrolled T2DM were included. Uncontrolled T2DM was determined by FBS value 129.6 mg/dl above or HbA1c value 7.2 mmol/L above [17, 20]. This study was conducted during the travel restrictions imposed by the government due to the COVID-19 pandemic; therefore, telephone interviews were conducted. Consequently, screening the participants with blood tests was not possible, and we recruited participants based on the values of FBS or HbA1c reported based on their recent blood reports. However, the majority of the participants were not aware of their HbA1c values. Therefore, participants who reported an FBS level of 130 mg/dl or above were considered as uncontrolled T2DM in this study. Additionally, patients who self-reported non-adherence to medicines and those who expressed willingness to articulate their experiences were included. Patients who had T1DM, hearing impairment, difficulty in communicating, and were disoriented to time, place and people were excluded. Participants were recruited until data saturation was reached. When the same information appears repeatedly in interviews, data saturation is achieved [21]. After completing 12 interviews, the authors found that there was no new information coming. Two additional interviews were conducted to check the data saturation. The data collection was completed with 14 interviews as no new codes were obtained with two additional interviews.

### Data collection

A semi-structured interview guide (S1 Appendix) designed by researchers based on the research objectives and available literature was used. A holistic conceptual framework model to describe medication adherence guided the questions of the interview guide [22].

The interview guide consisted of nine open-ended questions and was reviewed by two experts in pharmacy and nursing who had experiences in qualitative research and medication management. The interview guide was pre-tested among three older people from the general population for its face validity, including the readability and understandability of the items. Socio-demographics of participants, including age, sex, ethnicity, religion, marital status, level of education and working status, were collected before interviews.

The approval for the data collection, particularly the extraction of contact numbers of the potential participants, was obtained from the Director, Teaching Hospital, Peradeniya, Sri Lanka. Participants were recruited from the Diabetic Clinic of the Teaching Hospital, Peradeniya, Sri Lanka and the community during May 2021 to June 2021. The participants from the hospital clinic were recruited through telephone contact while the snowball sampling method was followed in community recruitment. Participants were interviewed over the telephone at their convenience time when they were at home. This study was conducted during the COVID-19 pandemic; therefore, this method was the most practical. The average duration for interviews was 33 minutes, ranging from 20–45 minutes. Informed verbal consent was obtained from each participant before data collection. All interviews were digitally recorded, including verbal consent, with prior permission from the participants. While conducting interviews, only the interviewer (SU) and the participant were present. Reflective notes were maintained after each interview.

### Data analysis

The digitally recorded files of the interviews were transcribed verbatim by the first researcher. The accuracy was checked by comparing it with the original recorded files. Demographic data were analysed using SPSS software (version 21). Inductive thematic analysis based on Braun and Clarke’s method [23] was used for qualitative data analysis. Two researchers coded the first three transcripts independently by active reading and re-reading of the transcripts. Consensus for the codes was achieved among the two researchers. Based on the coding of the three transcripts, an initial codebook was developed. The first researcher continued coding for the whole data set. The new codes that emerged were discussed with the second researcher to achieve consensus. Data were grouped into categories, themes and sub‐themes by the two researchers. Themes were reviewed, and definitions for each theme were built up. Naming for each theme was completed at the end of the data analysis. The findings were reported, and the consolidated criteria for reporting qualitative research (COREQ) checklist [24] was followed (S2 Appendix). In reporting, two researchers translated relevant quotes from Sinhala into the English language and consensus was achieved.

### Ethical considerations

The ethical clearance for this study was obtained from the Ethics Review Committee, Faculty of Allied Health Sciences, University of Peradeniya, Sri Lanka (No: AHS/ERC/2021/009). The Director, Teaching Hospital, Peradeniya granted permission to recruit participants from the Diabetic Clinic, mainly collecting contact details of the potential participants. Informed verbal consent was obtained from each participant, and the consent was audio recorded as a part of the interviews. In this study, any personal data were not collected (i.e., names). However, any personal identities in verbatim were removed following transcription and before data analysis.

### Trustworthiness of the study

The trustworthiness of the study was assured by several means, such as transferability, dependability and confirmability [25]. Transferability was confirmed by providing sufficient informative data on the setting, sample and sampling strategy, demographics, and semi-structured interviews. To assure dependability, the thematic analysis followed by Braun and Clarke’s method [23], the accepted standards of data analysis, was used. Confirmability was strengthened by developing a codebook based on the first three transcripts after achieving consensus among the two researchers for codes. The consensus was achieved for the newly formed codes in the transcription of other interviews. Consensus meetings were also held in the generation of themes and sub-themes.

## Results

### Participant characteristics

In this study, 14 older people with uncontrolled T2DM were interviewed, including eight males and six females (Table 1). The average duration of the interviews was 33 minutes, ranging from 20 minutes to 45 minutes.

**Table 1:** Socio-demographic data of participants (n = 14).

Characteristics of the participants	Number	Percentage (%)
**Age (in years)**		
61–65	5	35.71
66–70	4	28.57
71–75	3	21.43
76–80	2	14.29
**Gender**		
Male	8	57.14
Female	6	42.86
**Religion**		
Buddhism	13	92.86
Christian	1	07.14
**Marital status**		
Married	10	71.43
Widowed	4	28.53
**Education level**		
Illiterate	0	0
Primary education	0	0
Secondary education	12	85.71
Diploma	2	14.29
**Occupational status**		
Retired	7	50.00
Retired and self-employed	1	07.14
Self-employed	1	07.14
Employee	2	14.29
Household duties	3	21.43
**Living status**		
Living with a spouse and at least one adult offspring	5	35.71
Living with a spouse only	5	35.71
Living with at least one adult offspring	2	14.29
Living alone	2	14.29
**FBS**^**a**^ **(mg/dL)** *		
101–150 **	6	42.86
151–200	7	50.00
201–250	1	07.14
**The number of years of diabetes**		
1–5	2	14.29
6–10	3	21.43
11–15	4	28.57
16–20	2	14.29
21–25	3	21.43
**Self-reported medication use**		
OHAs ^b^ only	13	92.86
OHAs and Insulin	1	07.14

* Approximate values were considered because many of the participants were not able to report exact values.

** Lower limit for inclusion was 130 mg/dl

^a^ FBS

Fasting blood sugar

^b^ OHAs: Oral Hypoglycaemic Agents.

### Findings

Six key themes emerged from the interviews, including a theme related to the impact of COVID-19 on medication adherence, as this study was conducted during the COVID-19 outbreak (Table 2). In the following section, these six main themes with accompanying sub-themes are described with sample quotes. Participants were made anonymous using a participant number (e.g.: P1) in the reporting.

**Table 2:** Themes and sub-themes.

No	Themes	Sub-themes
1	Impact of knowledge, attitudes and practices on medication adherence	• Knowledge of the disease process • Knowledge of medications • Attitudes towards medication adherence • The practice of different treatment approaches • Self-medication and dosing
2	Treatment-related barriers to medication adherence	• Negative experiences related to medications • Polypharmacy
3	Impact of age-related changes on medication adherence	• Age-related memory impairment • Age-related visual disturbances • Age-related physical weaknesses
4	Person-related barriers to medication adherence	• Changes in lifestyle and roles • Influence of work-life • Motivation • Negligence
5	Impact of COVID-19 on medication adherence	• COVID-19-related guidelines imposed by the government • Healthcare system-related issues during the COVID-19 pandemic
6	Role of support systems in medication adherence	• Family support • Support and services received from health workers • Facilities available • Financial capability

#### Theme one: Impact of knowledge, attitudes and practices on medication adherence

This theme relates to the knowledge, attitudes and practices of participants’ medication adherence. Five sub-themes emerged as presented below.

*Knowledge of the disease process*. Participants were aware that uncontrolled DM could be caused by the consumption of more sugary foods. Some participants reported inadequate awareness of the normal and increased level of blood sugar, which had an indirect impact on medication adherence. For example, one participant stated, *“It cannot say that my sugar level is not in control because it is just around one hundred almost every time”* [P3]. Another participant stated that good knowledge about diabetes-related complications led to proper medication adherence. *“The increased sugar level is not good*. *It causes poor eyes and weak fingers*. *Sometimes wounds*, *will take so long to cure*. *Fortunately*, *I don’t have any*. *Because I take medicines*, *otherwise*, *I will get those"* [P13]. In contrast, some participants stated that they did not follow medicines properly because they did not take DM as a serious health problem. *“I did not take medicines*. *My father also had this disease*. *So I don’t mind it”* [P12].

*Knowledge of medications*. Poor knowledge of medications was highlighted. Although participants used medicines daily, a large number of participants were unable to name the medicines they took. Instead, participants recognised medicines by their external features like colour and shape. *“I take two from those big white tablets*, *one capsule*, *one from white round ones and aspirin”* [P9]. Even though, some participants knew the names of the medicines, almost all of them were not aware of the doses and/or strengths of their medicines *“I don’t know the dose I have to take*. *Anyway*, *I take two metformin tablets”* [P11].

Moreover, some participants believed that if they followed the diet properly without sugary foods, forgetting to take their medicines could have a minimal impact on DM management. Some participants stated that skipping a dose or missing one was not a big issue as long as they continued their treatment with the next dose. “*I am careful what I eat and drink*. *Forgetting to take medicines is fine as long as you are careful with your mouth”* [P7]. One participant reported that she intentionally skipped a dose of medicine because she hated taking them and that missing did not significantly affect sugar control. *“I hate taking medicines*. *Sometimes*, *I just feel I don’t want medicines today*. *So*, *I don’t take those*. *And also*, *since we take them every day*, *it doesn’t matter if you don’t take a single dose”* [P9].

*Attitudes towards medication adherence*. Ageing-related attitudes had negative impacts on medication adherence. For example, one participant believed that she did not want to take medicines as life was uncertain due to age. *"Now I am fed up with taking medicines for this long*. *It would be better if I could live freely in this older age*. *We don’t know when we will go”* [P9]. Some participants held negative attitudes towards medication, and they believed that Western medicine did not enable them to control sugar levels. One participant reported that she used herbal remedies to lower her blood sugar level as those remedies were more reliable than Western medicine. *“Yes*, *I am trying herbs*. *Earlier*, *I wanted to undergo an operation on one eye*. *It was stopped by doctors because my blood sugar level was high*. *Then I had ‘Thembu’ leaves (leaves of a local plant*: *Costus speciosus)*. *It reduced my sugar level*. *Then*, *I went for the operation in the following month*. *I have had leaves more than medicines*. *That’s how I reduce blood sugar level”* [P3].

Some participants highlighted the impact of the views of the general public on their medication adherence. For example, one participant said that *“Many people say that this disease can control if you control your diet*, *and it is the best way*, *not medicines at all*. *Therefore*, *I try to control this by controlling diet without taking pills”* [P9].

*The practice of different treatment approaches*. This study found that participants applied different treatment approaches or practices. Some participants stated that skipping or missing a dose was of little concern as long as they used alternative treatments. For example, one participant said, *“Sometimes I missed my pills*. *It doesn’t matter because I follow Ayurveda too”* [P12]. Participants further stated that traditional and herbal remedies were often used in conjunction with Western medicine. They believed that Ayurveda treatments would cure the root causes of the disease with fewer side effects; therefore, they did not want to take Western medicine for their whole lives. *“Taking pills for your whole life is not good for the body*. *Our kidneys can be damaged when we take many medicines like that*. *As you become older*, *it’s even harder*. *But Ayurveda medicines are like boiling leaves of trees and all*. *These natural things are not poisonous and you don’t have to keep taking medicines”* [P9]. Some participants have switched between Western and alternative medicines from time to time and skipped oral hypoglycaemic agents (OHAs) when taking traditional or herbal remedies. Moreover, they reported that they followed complementary and alternative medicines (CAMs) compared to prescribed medicines when the sugar level was high. One participant stated that the leaves of the ‘Kowakka’ plant (a herbal plant: *Coccinia grandis (L*.*) Voigt*) were used before checking blood sugar levels, and it helped to lower blood glucose levels in the reports, which prevents increasing the doses by the doctor. *“I take ‘Kowakka’ (Coccinia grandis (L*.*) Voigt) leaves before going for blood tests when I was not able to take medicines*. *Otherwise*, *the doctor may increase the doses unnecessarily every day”* [P9].

*Self-medication and dosing*. Some participants reported self-medication and dosing, for example, taking low doses of OHAs when sugar levels were normal. One participant said that she did not take a mid-day dose. *“Now*, *I need to take pills three times; morning*, *noon*, *and night*. *I drink only in the morning and evening*.*”* [P5] Another participant reported taking medicines from the nearest pharmacy without consulting a doctor. *“Some days*, *I could not go to the clinic at the right times*. *I took those from the nearest pharmacy*. *Really*, *I do not follow medicines properly”* [P14].

#### Theme two: Treatment-related barriers to medication adherence

This theme refers to treatment-related factors which affect medication adherence. Two sub-themes emerged as presented below.

*Negative experiences related to medications*. Most participants reported that taking medicines every day was a stressful experience, while some reported that taking medications frequently was an unpleasant experience. Moreover, some participants were worried that missing doses would result in increased blood sugar. *“Medicines help to control blood sugar*. *If I did not take my pills*, *there would be an increase in sugar level”* [P4].

Another common negative experience was the side effects of OHAs. Anorexia, nausea, and drowsiness were the most common side effects reported. Several participants reported that anorexia was a displeasing experience which led to avoid medicines. Another participant said that drowsiness interfered with his job performance; therefore, he did not take medicines. *“I feel sleepy when I take those pills*. *I am a security officer*. *I don’t like to lie to the job I do*. *So*, *I have nothing else to do; I don’t take medicines at night*. *How could I work if I am sleepy*? [P12].

Additionally, some participants highlighted that the side effects of Western OHAs were caused by their chemical basis. For example, one participant stated that *“English Medicines (a commonly used term referring to Western medicine) are chemicals*. *When you take more*, *it gives you another illness*. *Then*, *we have to take more medicines for such illnesses*. *The best way is avoiding those”* [P6].

*Polypharmacy*. Many participants reported that they presented with one or more comorbidities such as hypertension, hyperlipidaemia, heart diseases and arthritis. Therefore, the participants had to take several medicines (polypharmacy), which proved displeasing for them, resulting in poor medication compliance. *“When you have to take a lot of medicines like that for different illnesses*, *it is disgusting”* [P5].

#### Theme three: Impact of age-related changes on medication adherence

This theme refers to physiological changes associated with ageing, which affects medication adherence. Three sub-themes emerged as presented below.

*Age-related memory impairment*. This study found that age-related memory impairment was a significant barrier to medication adherence. One participant reported difficulties in remembering to take medicines. *“This is what happens when ageing*, *right*? *When my daughter asked whether I had taken medicines*, *I remembered that I had forgotten*. *Sometimes*, *I don’t remember whether I took pills or not actually”* [P7]. One participant stressed that he could not remember the time for taking medicines, and there is no one to remind him about the time for taking medicines.

*Age-related visual disturbances*. Many participants reported that age-related visual disturbances had a negative impact on medication adherence. One participant reported that the instructions written on medication covers could not be read well due to poor eyesight. Moreover, some participants reported that they could not read related educational materials available. One participant highlighted that although the hospital has set up various boards, notices and materials to educate patients on proper disease management strategies, it will not be effective as expected for visually impaired older people. *“The clinic has noticed what foods to eat and what to not*. *The importance of taking medicine*. *How the feet are examined*, *and the details are shown through pictures*. *But I have poor eyesight”* [P10].

*Age-related physical weaknesses*. Additionally, age-related physical weaknesses and dependency had a negative impact on medication adherence. For example, one participant reported that she could not go to the clinic because she was weak; therefore, she wanted help from others. *“I go to the clinic with my son by his motor bicycle*. *I cannot go alone because I am weak*. *It takes about an hour by bicycle to the clinic*. *If my son cannot get leave from his work*, *I wouldn’t be able to go to the clinic”* [P9]. Another participant reported that only the caretaker attended the clinic and queue to take medicines as he was very weak. Consequently, the information given by the pharmacist was not properly communicated to the patient. *“I don’t know; our daughter often goes to the queue as I cannot stand for a long time*. *She does not tell whether they told something special”* [P10].

#### Theme four: Person-related barriers to medication adherence

This theme refers to person-related factors which may affect medication adherence. Four sub-themes emerged as presented below.

*Changes in lifestyle and roles*. The life changes, particularly retirement, had an impact on medication adherence. The main reason was a sedentary lifestyle following retirement. *“Now*, *I am a retiree; after our daughter went to work*, *I used to stay in bed a little longer before breakfast*. *Most of the time*, *I fell asleep again*. *So*, *I wake up at noon*, *and then there is no morning meal or morning dose of medicines”* [P10]. One patient perceived that leaving her job caused cessation of usual physical activities, thereby causing exacerbations of DM. *“I take medicines*, *but they should be taken after meals*. *Since my retirement*, *I do not have adequate activities and do not feel hungry; I don’t take food on time”* [P14].

*The influence of work-life*. Although most participants were currently unemployed, some reported engaging in various works or occupations to earn their day-to-day expenses. They further noted that their work-life was related to medication non-adherence directly. For example, one participant said that he intentionally refrained from carrying medicines to the workplace as he usually forgot to take medicines when he worked. Another participant who is a security officer reported that he skipped taking medicines intentionally because he experienced sleepiness at the workplace after taking medicines. Moreover, some participants highlighted that they missed clinics due to their work commitments, resulting in medication non-adherence. One participant reported that he could not receive leave to attend the clinic. Another participant stated that he being scared to go to the clinic when he missed one clinic visit due to work matters. *“No*, *actually*, *I don’t have a problem with money*. *When I was off from work*, *when my duties were over*, *the doctor was not at the clinic*. *That’s the issue*. *When I missed the date*, *I felt scared to go on the following date”* [P11].

*Motivation*. One participant stated that seeing a relative with diabetes complications led to taking medicines regularly. Conversely, another participant stated that seeing his father dying with kidney failure due to diabetes medicines was a negative motivation towards following medicines regularly. *“I am afraid of taking these medicines because I know what happened to my father*. *He left us due to kidney failure due to using these pills for a longer time*. *I’m not saying I’m not afraid to die*. *It’s even harder to think that I*, *too*, *will die like this"* [P10].

*Negligence*. Many participants reported that they forgot to take medicines due to their negligence, and it was a significant barrier to medication adherence. Some participants stressed that their housekeeping roles interfered with medication-taking behaviour. *“I have to do all housekeeping at the house when my children go to work*. *Then I forget to take my pills*, *this is my fault”* [P6]. Some participants stated that they were not taking medicines during their stay outside. When they visit and stay with their children, relatives or friends, they often forget to take their medicines. *“When I go somewhere like my child’s place or one of my friend’s homes*, *I forget to take medicines”* [P4].

#### Theme five: Impact of COVID-19 on medication adherence

This additional theme highlights the impact of the COVID-19 pandemic on the medication adherence of people with DM. Two sub-themes emerged as presented below

*COVID-19-related guidelines imposed by the government*. The guidelines imposed by the government for controlling COVID-19 have influenced the management of chronic diseases like DM. One participant stated that she was unable to come home due to travel restrictions imposed by the government; therefore, she missed the medicines. *“I was in the child’s house*. *I could not return to my house due to Corona travel restrictions*. *I missed my pills”* [P13]. Another participant stated that he had to spend big money to take a taxi to go to the clinic as public transport was not available during the COVID-19 period. He further noted that it would be challenging to do every month. “*In the days of Corona*, *we went to the clinic by a taxi three-wheeler*. *It was too costly for us*. *As there was no public transport*, *we could not continue this every day*” [P10]. Another participant reported that he could not attend the clinic due to self-quarantine and missed free medicines issued by the government clinic.

*Healthcare system-related issues during the COVID-19 pandemic*. Participants highlighted the impact of COVID-19 on the healthcare delivery system. The main issue faced by participants was limited staff available in clinics and providing limited services. One participant stated that pharmacists only dispensed medicines during COVID-19 time and did not educate the patients. Therefore, they did not receive enough information “*Pharmacists do only give us medicines from that small counter due to COVID-19 restrictions; do not give instructions now”* [P6]. Moreover, one participant stated that some medicines were unavailable or out-of-stock in the nearest pharmacies, particularly small private pharmacies during the COVID-19 pandemic. Therefore, it was not easy to buy medicines when the amount given by the hospital was over. “*I struggled to find medicine because of Corona*. *The medicines I want to buy are usually not available in small pharmacies”* [P3].

#### Theme six: Role of support systems in medication adherence

This theme includes the positive and negative impacts of support systems on medication adherence among older people with DM. Four sub-themes emerged as presented below.

*Family support*. Some participants stated that their caretakers were busy, leading to medication non-adherence. *“My daughter looks after everything*, *not only our medications*. *But*, *they also have to do so much work for their lives*, *not only taking care of us*. *So*, *they could not stay at home every day and always*. *That is the problem”* [P7]. Another participant reported that family conflicts influenced medication adherence. *“When family problems arise*, *sometimes*, *quarrelling in the home*, *I wonder why I am taking medicines”* [P6]. Besides, some participants stressed the availability of positive family support. They reported that they had a good supportive environment at home, which helped them adhere to treatment. *“My daughters and sons help me in different ways*. *They give money to buy medicines*. *I follow medicine regularly”* [P3].

*Support and service received from health workers*. Another support mechanism reported was the support received from healthcare workers. However, some participants reported being scared to discuss with doctors when their sugar level was high. *“I’m scared; I’m not going to ask for advice from the doctor*. *I feel like I’m being blamed for too much sugar”* [P6]. Some participants reported that they wanted to know more about the blood tests and what medicines are used in DM, but they were scared to ask the health staff.

One participant highlighted that the time spent with each patient by health workers during clinic visits was not adequate to provide proper education. *“The doctors and nurses give us basic instructions to control our diet while taking medicines etc*., *but not in-depth*. *They do not talk with us for that much of a long time”* [P8]. Another participant reported that pharmacists did not teach adequately while dispensing the medications.

*Facilities available*. Furthermore, one participant stated that clinics are overcrowded; therefore, they needed more time to ask questions and clarify problems related to medication management. *“Clinics are overcrowded*. *There is not enough time to talk with staff in detail”* [P8]. One participant stated that the pharmacy counter is tiny, and they cannot ask anything from pharmacists. “*Oh no*! *You can’t ask for anything from the pharmacy*. *There’s not enough space from that small window”* [P6].

Although government sector clinics provide free medicines, participants stated that they had to purchase drugs from outside pharmacies due to the unavailability of medicines in government hospitals. Some participants reported difficulties in purchasing medicines from the nearest pharmacies because some drugs are not available as they are small pharmacies. *“That pill is not available in small pharmacies near to our place*. *I have to ask someone to buy medicines from a pharmacy in town*. *They are not available in small pharmacies nearby”* [P3].

*Financial capability*. Financial problems are an important factor highlighted by some of the participants. Some participants reported that they did not take medicines from time to time when they did not have enough money to buy medicines. Some participants said that they had to prioritise other everyday living expenses over medications. *“We have other expenses too*, *not only for medicines*. *Sometimes*, *I do not take medicines for days when it is difficult to buy them”* [P5]. One participant stated that he did not want to ask for money from children. Another participant said that he followed a private clinic when he was working. But, he missed that clinic as he retired and resorted to government hospitals due to unbearable medication costs in the private sector and channelling. Some retirees emphasised that their pension was inadequate to cover the cost of drugs.

## Discussion

In order to control diabetes mellitus and its complications, an appropriate medication regimen is essential [26], and medication adherence confirms the treatment success. Medication non-adherence is a common problem associated with managing chronic illnesses, particularly in older people. This study aimed to gain an in-depth understanding of medication adherence experiences among older people with uncontrolled T2DM and suggests that factors associated with medication non-adherence are not only patient-related but also encompass a broad spectrum of external factors.

### Impact of knowledge, attitudes and practices on medication adherence

This study reports that older people have inadequate knowledge of medicines which leads to poor medication adherence, confirming the previous study findings with the general population [27, 28]. In this study, many patients do not know or cannot recognise their medications’ names. Conversely, a study conducted in Malaysia reports that all patients know the names and doses of the medicines they use [29]. In the present study, people who know the names are also unaware of the doses or strengths of medications. Patients have also identified their medicines according to their external features. If the manufacturers change the external feature or the government decides to purchase medicines from a different manufacturer, there is a risk of taking medication incorrectly. Consequently, improving knowledge of medicines is essential to improve the safe use of medicines among older people with DM. Therefore, health professionals, particularly doctors, nurses and pharmacists, need to pay special attention to delivering adequate knowledge for the patients.

This study reports that patients’ attitudes toward the use of medicines are related to medication adherence among older people with DM. Some participants held that they were aged; therefore, they felt that they did not want to take medicines. Therefore, cognitive behavioural interventions [30] are essential to make awareness of the worth of life. Similar to the findings of a previous study [31], societal influence affects medication adherence; if acquaintances tell patients to follow complementary and alternative medicines (CAM) rather than taking Western medicine or trying to control the disease by diet alone, patients have motivated to follow them. The literature supports that sometimes views of society are more powerful [16] compared to the health professionals’ teaching. Additionally, some studies have reported that religious, spiritual or ritual beliefs drive medication adherence among people with DM [31]. When a wide range of attitudes and societal influences exists, patient-specific approaches must be initiated to improve the correct use of medications.

Similar to the previous studies [17, 27, 31], this study further reports that medication adherence is influenced by the use of various treatment methods, particularly CAMs while taking Western medicine. A majority of the participants are on at least one CAM; for example, they use herbal remedies and/or ayurvedic treatments. Although a previous study reports that some patients resort to CAMs due to the influence of acquaintances [31], the present study reports the voluntary use of CAMs. Moreover, this study reports that the participants prefer alternative treatments over Western medicine because they view traditional remedies are safe and efficient. These findings are consistent with previous studies [27, 31] while inconsistent with a recent study in Kuwait [28]. Interestingly, the present study reports that older people use CAMs, for example, herbal leaves to lower their FBS while taking Western medicine. This practice may lead to poor controlling the symptoms and the development of complications.

Moreover, this study reported self-medication and self-dose adjustment by older people with uncontrolled T2DM. These findings are consistent with previous studies [17, 29]. These problems lead to suboptimal or toxic effects of oral hypoglycaemic agents (OHAs). Consequently, health education on adherence to medications needs to be expanded. The development of strategies and research is recommended to record and monitor all types of medicines (i.e., Western, CAMs) used by the patients.

### Treatment-related barriers to medication adherence

Participants’ lived experiences with the disease interfere with medication adherence [16], and this study reports that the need for long-term medications leads to developing negative attitudes toward medication adherence. Therefore, education and adequate counselling are needed for patients who developed negative experiences with using medication for an extended period. Moreover, this study reports that frequent dosing leads to non-adherence. Therefore, reducing dosing frequency for older people with uncontrolled T2DM [32] or using sustained-release tablets where possible is recommended [33]. Additionally, the experience of side effects of OHAs was an essential factor in medication adherence among the study sample. The most commonly reported side effects are nausea, anorexia and drowsiness. Literature supports that side effects of medicines are associated with medication non-adherence [16]. The present study further reports that some patients are unaware of the side effects of OHAs. Similar findings have been reported previously [29]. Healthcare workers need to monitor the side effects of medications and can provide adequate information to fill the above knowledge gap.

Polypharmacy is a common problem among people with multiple chronic health problems. People with DM usually take five or more medicines to optimise blood glucose levels, blood pressure, and lipid control and manage other diabetes-related complications and comorbidities [32]. Consistent with previous studies, this study confirms that polypharmacy is a barrier to medication adherence among older patients with DM [31, 34]. Besides the possibility of medication non-adherence, polypharmacy causes several negative consequences, such as adverse drug effects and interactions [35, 36]. The Law of Therapeutic Parsimony highlights the need for using the minimum number of drugs, drug combinations, or drug preparations as well as minimum doses and frequency to achieve pre-decided therapeutic outcomes [37]. Consequently, a multi-disciplinary approach to minimising the impact of polypharmacy in older people with DM is needed [35].

### Impact of age-related changes on medication adherence

The normal ageing process is associated with a decline in cognitive functions [38], and literature supports that memory plays a distinct role in medication adherence [39]. This study reveals that age-related memory impairment attributes to medication non-adherence in older people with uncontrolled T2DM. Therefore, strengthening family support is essential and additional support systems should be initiated and strengthened to minimise the impact of memory impairment in medication non-adherence among older people with uncontrolled T2DM. Moreover, older people’s poor eyesight is a barrier in proper medication management [40]. The present study also supports this phenomenon; therefore, health professionals need to pay special attention to the visual problems of older people, especially when providing information and health education. Additionally, poor vision can be developed as a complication of DM; therefore, monitoring visual problems, arranging eye referrals [20] and helping them to select appropriate visual aids are essential.

Age-related functional disabilities and weaknesses of older people result in physical dependency. The present study reports that the physical dependence of older people hinders proper clinic follow-up and meeting with health professionals. Therefore, flexible clinic schedules and strengthening support systems, particularly family support, are needed for older people with DM who are presented with age-related physical weaknesses.

### Person-related barriers to medication adherence

The present study reports that changes in lifestyle and roles affect medication adherence. As people transition to retirement, their lifestyles are likely to change dramatically, and literature supports that retirement is associated with reduced physical activity [41]. This study indicates that some retired older people report long sleeping times and lack of hunger, leading to medication non-adherence. Therefore, modifications of lifestyles and strategies to improve their activities in retirement life are essential. In line with a previous study [16], this study reports that housekeeping roles significantly reduced medication adherence among older people with uncontrolled T2DM. The patient-centred healthcare system needs to be shifted to family-centred approaches [42]. Although the study population was older people, some of them are still doing some jobs. Literature supports that medication adherence is challenging with working conditions [17, 31]. The present study confirms that patients struggle to take medications properly while working. This study further reports that distress towards asking for leaves from administrators and working away from homes negatively affected attending clinics and taking continuous medications. Therefore, proper education and counselling are essential for older people with uncontrolled DM who are currently working.

Additionally, this study reported that lack of motivation and negligence are associated with medication adherence. Negligence is accompanied by forgetfulness, staying outside the home and travelling. The impact of patients’ negligence on medication adherence has also been reported in previous studies [17, 27, 29, 43]. The literature further supports that forgetfulness is one of the main barriers to medication adherence among patients with DM [43–45]. Forgetfulness would be addressed through adherence strategy education, for example setting up alarms [44].

### Impact of COVID-19 on medication adherence

This additional theme has emerged concerning the COVID-19 outbreak. The government has imposed various COVID-19-related guidelines, for example, quarantining, travel restrictions and lockdowns, to minimise the spread of COVID-19 [46]. These guidelines have created additional challenges, particularly medication management and adherence for older people with uncontrolled T2DM. A similar situation has been reported in a recent study [47]. Minimising human movement which is an essential strategy in preventing the spread of COVID-19 [46], has negative impacts on attending clinics and getting prescription refills [47]. Patients with chronic diseases like DM and older people are at high risk of having more detrimental effects and dying from COVID-19 [47, 48]. Hence, special attention should be given to the above groups to ensure proper medication adherence during the pandemics like COVID-19. Additionally, with limited or no public transport, patients had to hire taxis to attend clinics, and this was costly for patients. Therefore, additional financial support or alternative transport facilities need to be initiated during a pandemic like COVID-19 to support people with poor economic status.

This study reports that government clinic followers had to take some medications from outside pharmacies, but medicines were out-of-stock in private pharmacies. During the COVID-19 pandemic, many low-to-middle-income countries have found a considerable gap in drug importation, especially for chronic illnesses [47]. These findings raise the need for policies to ensure the continuous availability of essential medicines equally during a crisis. Additionally, this study reports that patient education, awareness and counselling programs are not functioning correctly as a result of the COVID-19 situation. Consequently, patients have received limited education that directly impacts their medication adherence. Therefore, alternative methods, such as teleconsultation approaches (e-mail, online chat and video) [49], can be used in health education.

### Role of support systems in medication adherence

Supportive family environments were found to be helpful in medication adherence [1, 27, 29, 50]. The present study confirms that non-supportive family environments and family conflicts lead to medication non-adherence. Interactive and economical family support can improve medication adherence among people with DM [16]. Therefore, it is recommended to address older people and their family members to provide insights into the importance of family cooperation in medication adherence.

In the present study, doctors, nurses and pharmacists have been identified as the main healthcare providers who first contact patients and provide education and support. However, limited patient-care provider interaction and limited time availability are reported. Patient-provider communication is crucial in medication adherence in patients with DM [17]. Moreover, this study reports negative experiences related to clinics, for example, crowded clinics, taking a long time at clinics, and unavailability of medicines at clinics. Easy accessibility [51] and patient satisfaction [51] with healthcare facilities are pivotal in medication adherence. Consequently, developing institutional policies and guidelines is essential to ensure proper patient-provider interaction and system-related issues.

## Strength and limitations

To the authors’ knowledge, this was the first reported study to explore an in-depth understanding of experiences and barriers to medication adherence among older people with uncontrolled T2DM who followed government hospital clinics in Sri Lanka. We have used the qualitative approach that helps to gain an in-depth understanding of the research interest. Additionally, we achieved data saturation in data collection. Limitations of this study include not receiving transcripts for member checking and the inclusion of only Sinhala-speaking older people in the study. Additionally, we included participants who self-reported non-adherence to medication. To screen medication non-adherence, screening tools, for example, Morisky Medication Adherence Scale [52] can be used.

## Conclusion

Poor knowledge, practices and negative attitudes of patients towards medication adherence are some significant factors causing medication non-adherence among older people with DM in the Sri Lankan community. Other factors include treatment-related barriers, age-related changes, person-related barriers and unsupportive systems within and outside the healthcare facilities. Age-related physical and psychological changes, such as visual impairment, physical weaknesses and memory impairment, and ageing attitudes, negatively affect medication adherence among older people with DM. The trend of resorting to CAMs hinders medication adherence in older people. Moreover, the COVID-19 pandemic has added additional challenges. Consequently, support systems need to be strengthened. The need for implementing individualised patient teaching for older people with uncontrolled T2DM to improve medication adherence is timely. Expanding the role of healthcare professionals (pharmacists, nurses and doctors) is essential, especially in teaching patients. Educational and counselling programmes for older people with DM living in the community need to be expanded. In future studies, healthcare providers and family members/carers can be interviewed to explore this problem further. As medication non-adherence is a prevalent issue among older patients with DM living in the community in Sri Lanka, the assessment of the prevalence of this issue is recommended.

## Supporting information

S1 Appendix Interview guide. (DOCX)

S2 Appendix COREQ checklist. (DOCX)
