# Supplementary material for: Perspectives of older people with uncontrolled type 2 diabetes mellitus towards medication adherence: A qualitative study
Source: PLoS One. 2023 Aug 10;18(8):e0289834. doi: 10.1371/journal.pone.0289834 (PMC10414664; doi:10.1371/journal.pone.0289834)
Supplement: S1 Appendix — (DOCX) [file pone.0289834.s001.docx]

**S1 Appendix**

**Interview Guide**

| **Participant introduction** |
| --- |
| 1. Could you please tell me about yourself?   Probe:   - Self-introduction - Role in the family/family responsibilities |
| **Introductory questions** |
| 1. Could you please tell me about your history of diabetes?   Probe:   - - Starting date/diagnosed date   - Stage of diabetes/severity  1. Could you please tell me about the oral medications currently taking for diabetes?   Probe:   - Names, dose, time etc. - Starting date/month |
| **Experiences** |
| 1. Could you please tell me your experiences when taking oral medications for diabetes?   Probe:   - Difficulties in taking medications as prescribed by a doctor - Physical difficulties - Psychological difficulties - Availability of drugs - Medication cost - Family/social support - Myths |
| **Barriers to medication adherence** |
| 1. Could you please tell me the importance of the medications in managing your diabetic condition?   Probe:   - Importance of medications in the management of diabetes - Adequacy of knowledge to take medication continuously - Knowledge of side effects - Knowledge provider: Doctor, Nurse, Pharmacist, Other methods  1. Could you please tell me what type of barriers you encountered while taking medications to manage your diabetic problems?   Probe:   - Demographic factors (gender, age, education, marital status, ethnicities, financial status) - Disease-related factors (duration of disease, level of HbA1C) - Medication regimen related factors (types of medications, number of medications, complexity, duration of treatment, side effects, polypharmacy, method of administration) - Healthcare provider related factors (knowledge, attitudes and beliefs of care provider, communication issues, shared decision making) - System-related factors (availability of resources, financial issues – insurance, cost of medicines and services, care process- visit duration, continuity, strict guidelines, availability of translators) - Societal-related factors (culture - beliefs, perceptions, social support, stigma, vicarious experiences) |
| 1. Could you please tell me, how do you think that you can improve taking medications regularly? What are the strategies that you can follow?   Probe:   - The solution you think to work for you |
| 1. Is there anything else you would like to add to our understanding of taking medications regularly? |
| 1. Do you have any questions or further responses? |
