# Supplementary material for: Perspectives of older people with uncontrolled type 2 diabetes mellitus towards medication adherence: A qualitative study
Source: PLoS One. 2023 Aug 10;18(8):e0289834. doi: 10.1371/journal.pone.0289834 (PMC10414664; doi:10.1371/journal.pone.0289834)
Supplement: S2 Appendix — (DOCX) [file pone.0289834.s002.docx]

S2 Appendix

Consolidated criteria for reporting qualitative research (COREQ): a 32-item checklist

Developed from:

Tong A, Sainsbury P, Craig J. Consolidated criteria for reporting qualitative research (COREQ): A 32-item checklist for interviews and focus groups. *Int J Qual Heal Care*. 2007;19(6):349-357. doi:10.1093/intqhc/mzm042

| **No Item** | **Guide questions/description** | **Page No** |
| --- | --- | --- |
| **Domain 1: Research team and reflexivity** | | |
| Personal Characteristics | | |
| 1. Interviewer/facilitator | Which author/s conducted the interview or focus group? | 6  Main document |
| 1. Credentials | What were the researcher’s credentials? E.g. PhD, MD | Title page |
| 1. Occupation | What was their occupation at the time of the study? | Title page |
| 1. Gender | Was the researcher male or female? | Title page |
| 1. Experience and training | What experience or training did the researcher have? | Title page |
| Relationship with participants | | |
| 1. Relationship established | Was a relationship established prior to study commencement? | 6  Main document |
| 1. Participant knowledge of the interviewer | What did the participants know about the researcher? e.g. personal goals, reasons for doing the  research | 6  Main document |
| 1. Interviewer characteristics | What characteristics were reported about the interviewer/facilitator? e.g. Bias, assumptions, reasons and interests in the research topic | 6  Main document |
| **Domain 2: study design** | | |
| Theoretical framework | | |
| 1. Methodological orientation and Theory | What methodological orientation was stated to underpin the study? e.g. grounded theory, discourse analysis, ethnography, phenomenology, content analysis | 4  Main document |
| Participant selection | | |
| 1. Sampling | How were participants selected? e.g. purposive, convenience, consecutive, snowball | 5  Main document |
| 1. Method of approach | How were participants approached? e.g. face-to-face, telephone, mail, email | 5  Main document |
| 1. Sample size | How many participants were in the study? | 8  Main document |
| 1. Non-participation | How many people refused to participate or dropped out? Reasons? | 5  Main document  (those who expressed willingness to articulate their experiences were included in the study, therefore no one refused) |
| Setting | | |
| 1. Setting of data collection | Where was the data collected? e.g. home, clinic, workplace | 4  Main document |
| 1. Presence of non-participants | Was anyone else present besides the participants and researchers? | 6  Main document  (While conducting interviews, only the interviewer (SU) and the participant were present) |
| 1. Description of sample | What are the important characteristics of the sample? e.g. demographic data, date | 9  Table 1 |
| Data collection | | |
| 1. Interview guide | Were questions, prompts, guides provided by the authors? Was it pilot tested? | 5  Main document |
| 1. Repeat interviews | Were repeat interviews carried out? If yes, how many? | No |
| 1. Audio/visual recording | Did the research use audio or visual recording to collect the data? | 6  Main document |
| 1. Field notes | Were field notes made during and/or after the interview or focus group? | 6  Main document |
| 1. Duration | What was the duration of the interviews or focus group? | 7  Main document |
| 1. Data saturation | Was data saturation discussed? | 5  Main document |
| 1. Transcripts returned | Were transcripts returned to participants for comment and/or correction? | 30  Main document |
| **Domain 3: analysis and findings** | | |
| Data analysis | | |
| 1. Number of data coders | How many data coders coded the data? | 6  Main document |
| 1. Description of the coding tree | Did authors provide a description of the coding tree? | No  Themes and sub-themes were given in Table 2 (10, main document) |
| 1. Derivation of themes | Were themes identified in advance or derived from the data? | 6  Main document |
| 1. Software | What software, if applicable, was used to manage the data? | Qualitative data analysis was done manually. Demographic data analysis: 6  Main document |
| 1. Participant checking | Did participants provide feedback on the findings? | No |
| Reporting | | |
| 1. Quotations presented | Were participant quotations presented to illustrate the themes / findings? Was each quotation identified? e.g. participant number | 10-22  Main document |
| 1. Data and findings consistent | Was there consistency between the data presented and the findings? | 7-22  Main document |
| 1. Clarity of major themes | Were major themes clearly presented in the findings? | Yes  9-22  Main document |
| 1. Clarity of minor themes | Is there a description of diverse cases or discussion of minor themes? | Yes  22-29  Main document |
